# Protective Roles of Grass Carp *Ctenopharyngodon idella* Mx Isoforms against Grass Carp Reovirus

**DOI:** 10.1371/journal.pone.0052142

**Published:** 2012-12-14

**Authors:** Limin Peng, Chunrong Yang, Jianguo Su

**Affiliations:** 1 College of Animal Science and Technology, Northwest A&F University, Shaanxi Key Laboratory of Molecular Biology for Agriculture, Yangling, Shaanxi, China; 2 College of Veterinary Medicine, Northwest A&F University, Yangling, Shaanxi, China; Auburn University, United States of America

## Abstract

**Background:**

Myxovirus resistance (Mx) proteins are crucial effectors of the innate antiviral response against a wide range of viruses, mediated by the type I interferon (IFN-I) signaling pathway. However, the antiviral activity of Mx proteins is diverse and complicated in different species.

**Methodology/Principal Findings:**

In the current study, two novel Mx genes (CiMx1 and CiMx3) were identified in grass carp (*Ctenopharyngodon idella*). CiMx1 and CiMx3 proteins exhibit high sequence identity (92.1%), and low identity with CiMx2 (49.2% and 49.5%, respectively) from the GenBank database. The predicted three-dimensional (3D) structures are distinct among the three isoforms. mRNA instability motifs also display significant differences in the three genes. The spatial and temporal expression profiles of three *C. idella* Mx genes and the IFN-I gene were investigated by real-time fluorescence quantitative RT-PCR (qRT-PCR) following infection with grass carp reovirus (GCRV) *in vivo* and *in vitro*. The results demonstrated that all the four genes were implicated in the anti-GCRV immune response, that mRNA expression of Mx genes might be independent of IFN-I, and that CIK cells are suitable for antiviral studies. By comparing expression patterns following GCRV challenge or poly(I:C) treatment, it was observed that GCRV blocks mRNA expression of the four genes. To determine the functions of Mx genes, three CiMx cDNAs were cloned into expression vectors and utilized for transfection of CIK cells. The protection conferred by each recombinant CiMx protein against GCRV infection was evaluated. Antiviral activity against GCRV was demonstrated by reduced cytopathic effect, lower virus titer and lower levels of expressed viral transcripts. The transcription of IFN-I gene was also monitored.

**Conclusions/Significance:**

The results indicate all three Mx genes can suppress replication of grass carp reovirus and over-expression of Mx genes mediate feedback inhibition of the IFN-I gene.

## Introduction

Viruses are the most abundant pathogens on earth and resistance of cells to them depends on their capacity to detect and control viral replication [1]. Host antiviral responses are initiated through the detection of viral components by host pattern recognition receptors (PRRs). Upon recognition, PRRs trigger signaling that results in expression of type I interferons (IFN-Is), IFN-stimulated genes, and infammatory cytokines that suppress viral replication and facilitate adaptive immune responses [2], [3]. IFN responses play a central role in the host antiviral defence [3]. IFNs are also essential innate antiviral components that protect fish from virus infection [4]–[6]. IFN-Is secreted by infected cells promote antiviral state in neighbouring cells by the induction of numerous antiviral genes [7]. Since the discovery of IFNs, considerable progresses have been made in describing the natures of the cytokines themselves and the signaling components that direct the cell responses and the antiviral activities. Gene targeting studies have distinguished four main effector pathways of the IFN-mediated antiviral responses: the Mx GTPase pathway; the 2′, 5′-oligoadenylate-synthetase-directed ribonuclease L pathway; the protein kinase R pathway; and the ISG15 ubiquitin-like pathway [8]. Among the known IFN-induced antiviral mechanisms, the Mx pathway is one of the most powerful [9].

Mx proteins have been discovered in many animals. They are IFN-induced dynamin-like GTPases, which are highly conserved in invertebrates and vertebrates [10]. Mx proteins consist of three domains: an N-terminal dynamin domain (containing dynamin family signature and tripartite GTP-binding motifs, DYNc); a central interactive domain (CID) mediating selfassembly; and a C-terminal GTPase effector domain (GED) (containing leucine zipper motif (LZ)) [11]. Mx proteins form homo-oligomers and self-assemble into ring-like and helical structures, which are critical for GTPase activity, protein stability, and viral recognition [11]. The homo-oligomerization, and therefore the antiviral activity, is the result of binding between the LZ region of an Mx molecule and the CID domain of a second neighbouring molecule [12]. They inhibit a wide range of viruses by blocking an early stage of the viral replication cycle [13], [14]. In general, Mx GTPases appear to detect viral infection by sensing nucleocapsid-like structures. As a consequence, these viral components are trapped and sorted to locations where they become unavailable for the generation of new virus particles [11]. Mx proteins can localize either in the cytoplasm or in the nucleus, thus allowing the inhibition of the virus replication cycle in different phases [15].

Mx proteins usually appear in different isoforms. Two Mx genes have been reported in amphioxus and human, whereas three have been described in the rat [16]. Interestingly, large inter-specific variability in the number of teleost Mx isoforms (from 1 to 7) has been disclosed: seven in *Danio rerio*; five in *Ictalurus punctatus*; three in *Oncorhynchus mykiss*, *Salmo salar*, *Oplegnathus fasciatus* and *Sparus aurata*; two in *Hippoglossus hippoglossus*, *Carassius auratus*, *Epinephelus coioides* and *Scophthalmus maximus*; and just one in *Perca fluviatilis*, *Gobiocypris rarus*, *Paralichthys olivaceus*, *Takifugu rubripes*, *Solea senegalensis*, and *Lates calcarifer*, etc [7], [17]. This large diversity supports the emerging role of the innate immune system variability in the defensive strategies of fish and lower vertebrates against pathogens.

The direct antiviral activity of Mx proteins was demonstrated more than a decade ago [18]. Since then, functional studies have especially focused on host-virus interactions, mostly in birds and mammals. However, not all Mx proteins have antiviral function. No antiviral function is detected in human Mx2 [19], rat Mx3 [20] or duck Mx [21].

Grass carp *Ctenopharyngodon idella* is one of the most important aquaculture species in China; its output reached 4.22 million tons in 2010, accounting for 18% of freshwater aquaculture production in China. Farmers suffer severe economic losses annually due to mortalities resulting from grass carp hemorrhagic disease, caused by grass carp reovirus (GCRV), which is classified taxonomically in the genus *Aquareovirus*, family *Reoviridae*. Better understanding of the immune defense mechanisms against the virus may contribute to the development of management strategies for disease control and long term sustainability of grass carp farming.

Only one Mx isoform (CiMx2) has been reported in grass carp and its antiviral activity remains unclear [22]. The current study was aimed at novel isoform exploration and functional characterization of the Mx proteins in grass carp to improve understanding of the natural resistance of this species to GCRV infection.

## Results

### 1 Identification of grass carp Mx cDNAs

Two additional full-length cDNA sequences similar to CiMx2 were identified by EST analyses and RACE techniques, and confirmed by PCR and sequencing. The two Mx isoforms were as follows: the CiMx1 gene consisted of 2881 bp, encoding a protein of 635 amino acids; and the CiMx3 gene was composed of 3002 bp, encoding a protein of 630 amino acids. The cDNA sequences were deposited in GenBank under accession number **HQ245104** for CiMx1, and **HQ839769** for CiMx3. The corresponding protein IDs were assigned **ADU33870** and **ADZ44601**, respectively.

To compare the features of the three grass carp Mx cDNAs, grass carp Mx2 cDNA sequence (accession No. **AY395698**) was retrieved from GenBank database. An mRNA instability motif (ATTTA) in the 3′ UTR was found in all the three Mx cDNA sequences, but the number of motifs differed: two motifs were found in CiMx1; three in CiMx2; and seven in CiMx3.

### 2 Homology analysis of deduced protein sequences

**Figure 1:**
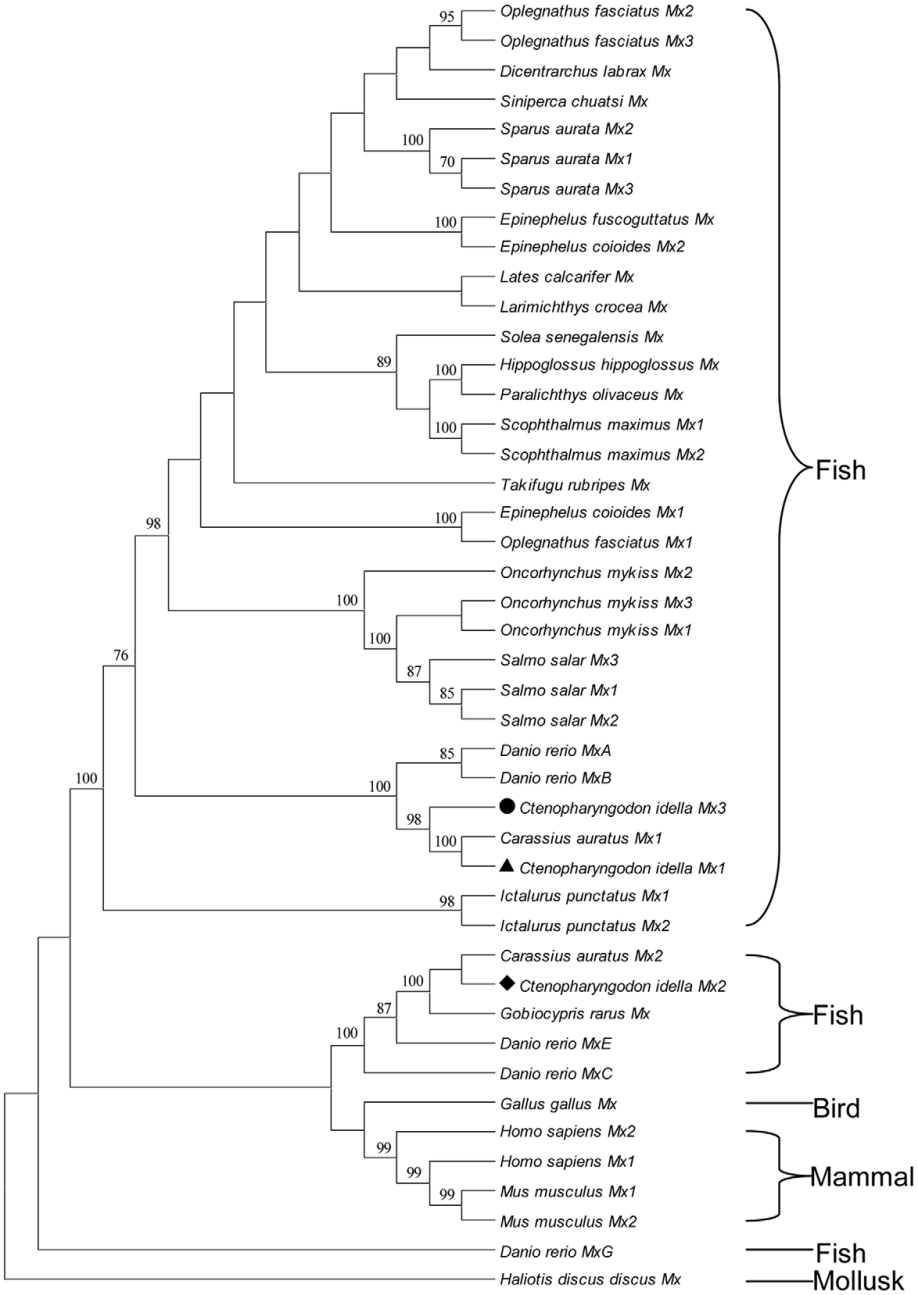
Phylogenetic relationships of all the fish, chicken, mouse and human Mx protein sequences in GenBank (***Danio rerio*** **MxD and MxF sequences are not included due to just partial sequences available**)**.** Maximum-likelihood phylogenetic tree generated from a MAFT alignment and MEGA 5.1 program. *Haliotis discus discus* Mx was employed as the outgroup. The bar indicates the distance. CiMx1, CiMx2 and CiMx3 were marked with triangle (▴), diamond (♦) and circle (•), respectively. The protein IDs are as follows: *Ctenopharyngodon idella* Mx1 **ADU33870**, Mx2 **AAQ95584**, Mx3 **ADZ44601**; *Carassius auratus* Mx1 **AAP68828**, Mx2 **AAP68827**; *Danio rerio* MxA **NP_891987**, MxB **Q800G8**, MxC **NP_001007285**, MxE **NP_878287**, MxG **CAD67761**; *Dicentrarchus labrax* Mx **AAR99718**; *Epinephelus fuscoguttatus* Mx **ADE80885**; *Epinephelus coioides* Mx1 **ABD95979**, Mx2 **ABD95982**; *Gobiocypris rarus* Mx **ABL61237**; *Gallus gallus* Mx **CAA80686**; *Homo sapiens* Mx1 **NP_001171517**, Mx2 **NP_002454**; *Hippoglossus hippoglossus* Mx **AAF66055**; *Ictalurus punctatus* Mx1 **Q7T2P0**, Mx2 **AAY33864**; *Lates calcarifer* Mx **AAW22002**; *Larimichthys crocea* Mx **ABJ56003**; *Mus musculus* Mx1 **NP_034976**, Mx2 **NP_038634**; *Oplegnathus fasciatus* Mx1 **ACF75866**, Mx2 **ACF75867**, Mx3 **ACF75868**; *Oncorhynchus mykiss* Mx1 **AAA87839**, Mx2 **AAC60214**, Mx3 **AAC60215**; *Paralichthys olivaceus* Mx **BAC76769**; *Scophthalmus maximus* Mx1 **AAT57877**, Mx2 **AAT57878**; *Sparus aurata* Mx1 **ACK99554**, Mx2 **ACK99553**, Mx3 **ACN22085**; *Salmo salar* Mx1 **AAB40994**, Mx2 **AAB40995**, Mx3 **AAB40996**; *Solea senegalensis* Mx **AAV49303**; *Siniperca chuatsi* Mx **AAQ91382**; *Takifugu rubripes* Mx **AAO37934**; *Haliotis discus discus* Mx **ABI53802**.

To study the molecular evolution and compare sequence homology, we selected all Mx protein sequences from fish, chicken, mouse and human in GenBank and constructed a phylogenetic tree (Fig. 1). Piscine Mx sequences formed three branches, separated by mammalian and bird groups. Three grass carp Mx isoforms were located in two different groups, which were divided by mammalian and bird groups. The observations also supported that CiMx1 is closely related to CiMx3.

To further compare the sequence homology, the percent amino acid identities of several fish and human deduced Mx protein sequences are presented in Table 1. CiMx1 and CiMx3 showed the highest identities with *C. auratus* Mx1 (99.5% and 91.7%, respectively). CiMx2 showed the highest identity (89.8%) with *C. auratus* Mx2 [23]. The identities among the three grass carp sequences ranged from 49.2% to 92.1%, which was lower than those with three Mx members in one species.

**Table 1:** Sequence identities of the deduced amino acid sequences of Mx genes among fishes and human with serial members.

*Mx* protein	1	2	3	4	5	6	7	8	9	10	11	12	13	14	15	16	17	18	19	20	21	22	23	24	25	26	27	28	29	30
1. *C. idella* Mx1																														
2. *C. idella* Mx2	49.2																													
3. *C. idella* Mx3	92.1	49.5																												
4. *C. auratus* Mx1	99.5	49.1	91.7																											
5. *C. auratus* Mx2	49.5	89.8	48.5	49.2																										
6. *O. fasciatus* Mx1	59.8	46.7	59.8	59.4	46.6																									
7. *O. fasciatus* Mx2	72.6	50.8	72.4	72.1	51.4	67.9																								
8. *O. fasciatus* Mx3	71.1	50.9	71.5	70.6	50.7	67.6	91.2																							
9. *S. aurata* Mx1	71.2	52.1	71.9	70.7	52.1	66.6	88.2	87.4																						
10. *S. aurata* Mx2	71.3	50.5	70.9	70.8	50.2	66.0	87.0	86.1	93.1																					
11. *S. aurata* Mx3	72.0	51.5	72.5	71.5	51.2	67.8	88.8	87.4	95.7	93.0																				
12. *E. coioides* Mx1	64.4	48.9	63.5	63.9	49.5	73.5	72.2	73.2	71.4	71.5	71.9																			
13. *E. coioides* Mx2	71.2	53.0	71.4	70.7	52.4	67.2	87.7	86.5	85.9	85.3	86.1	72.3																		
14. *S. maximus* Mx1	68.7	50.3	68.7	68.2	50.5	63.5	82.9	80.3	80.4	79.5	80.4	68.4	82.4																	
15. *S. maximus* Mx2	68.3	50.0	68.4	67.9	50.2	63.4	82.6	79.9	80.0	79.2	80.0	68.1	82.1	99.4																
16. *O. mykiss* Mx1	73.9	51.8	73.0	73.4	52.0	65.5	82.3	81.2	82.0	79.7	81.4	70.8	81.0	77.0	76.7															
17. *O. mykiss* Mx2	72.6	51.5	71.0	72.1	51.2	63.0	79.2	78.5	78.0	78.3	78.3	69.4	78.3	74.4	74.1	86.5														
18. *O. mykiss* Mx3	74.1	51.3	74.0	73.6	52.1	65.8	82.5	81.4	81.4	79.7	81.3	70.1	81.0	77.5	77.2	96.3	86.3													
19. *S. salar* Mx1	74.0	51.5	73.5	73.5	52.3	65.5	82.5	81.1	81.5	79.6	81.4	70.4	80.9	77.8	77.5	96.0	86.5	97.9												
20. *S. salar* Mx2	73.5	51.3	73.2	73.1	52.2	65.1	82.0	80.6	81.1	79.1	80.9	69.8	80.4	77.7	77.4	95.3	85.8	97.3	99.4											
21. *S. salar* Mx3	74.8	51.5	74.1	74.3	51.9	65.6	82.5	81.4	82.1	80.3	81.9	70.5	80.9	78.0	77.7	96.3	86.5	95.8	97.1	96.5										
22. *D. rerio* MxA	88.3	48.8	86.9	88.0	49.4	58.8	72.0	69.9	71.2	71.1	71.2	62.9	71.7	68.2	67.9	72.2	71.5	72.8	72.8	72.3	72.5									
23. *D. rerio* MxB	84.7	48.7	86.0	84.3	48.8	59.2	71.2	71.8	71.8	70.5	71.9	64.2	71.7	67.9	67.4	73.0	70.1	72.9	72.7	72.2	73.4	85.3								
24. *D. rerio* MxC	47.6	68.9	47.3	47.5	69.8	44.6	48.7	47.3	49.1	48.0	48.9	46.8	48.5	47.7	47.4	49.1	48.5	48.8	48.8	48.8	48.6	48.1	47.1							
25. *D. rerio* MxE	49.3	79.6	48.8	49.0	80.4	47.2	51.6	50.5	52.3	50.5	51.6	49.8	51.6	49.8	49.7	51.2	50.7	51.1	51.4	51.3	51.2	49.1	48.4	74.8						
26. *D. rerio* MxG	43.5	43.1	43.0	43.5	42.9	40.0	43.6	43.3	44.2	42.9	44.0	41.8	44.7	43.3	43.0	45.7	44.7	45.4	45.7	45.8	45.8	42.3	43.7	42.7	43.8					
27. *I. punctatus* Mx1	71.1	48.8	70.9	70.7	49.0	61.8	74.1	72.6	73.9	72.4	73.1	65.7	74.2	70.4	70.1	74.5	72.9	75.4	75.4	74.8	75.6	71.0	70.4	47.5	49.5	43.7				
28. *I. punctatus* Mx2	62.7	47.9	62.5	62.5	47.4	55.4	64.8	64.5	64.5	63.2	63.6	59.6	64.4	62.5	62.7	65.7	64.2	65.9	65.7	65.2	65.4	62.9	62.2	45.3	47.7	41.2	74.6			
29. *H. sapiens* Mx1	52.1	49.8	51.6	51.8	49.8	47.4	53.8	52.8	53.8	53.0	54.0	51.7	53.1	52.8	52.4	52.6	52.1	52.3	52.4	52.3	52.7	51.6	50.2	47.8	50.5	43.2	51.4	49.2		
30. *H. sapiens* Mx2	45.7	46.3	44.2	45.6	44.8	43.5	45.6	45.7	46.4	45.5	46.6	45.3	46.3	45.6	45.2	45.3	45.2	46.2	46.1	46.0	45.4	45.3	44.9	43.1	45.1	40.2	45.4	43.6	57.0	

Note: The protein IDs are as Fig. 1. Although five Mx isoforms were described in *I. punctatus* in the text, they were based on five promoters identified. Actually, they are just two mRNA sequences deposited in GenBank, employed in the present comparison. *H. hippoglossus* appears to possess two Mx loci, as suggested by southern blot analysis of genomic DNA. In fact, only single sequence is retrieved in GenBank. So they were not exhibited in the current analysis.

**Figure 2:**
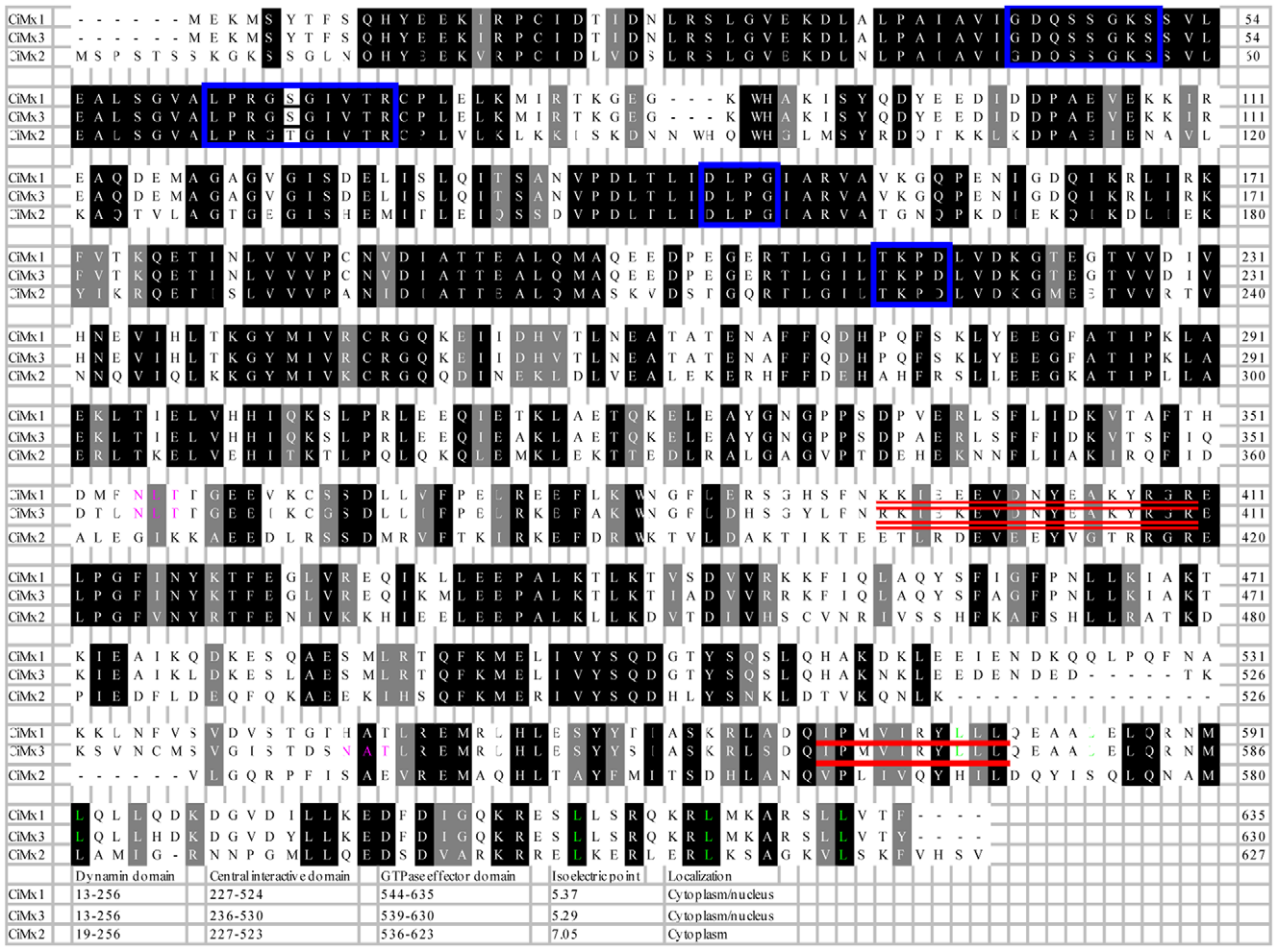
Alignments and characterizations of deduced amino acid sequences of grass carp Mx isoforms. Identical amino acids are in black background, and similar amino acids are in dark gray background. Tripartite GTP-binding motif consensus elements (GXXXSGKS/T, DXXG and T/NKXD), dynamin family signature (LPRG(S/K)GIVTR) are in blue boxes. The leucine residues of the LZ are shown in green. The potential N-linked glycosylation sites (NXT/S) are in pink. The bipartite nuclear localization signals ((K/R)(K/R)X_10–12_(K/R)_3/5_) are double lined. The nuclear export signals (L/I/V/F/M)X_3_(L/I/V/F/M)X_2_(L/I/V/F/M)X(L/I/V/F/M) are underlined. The positions of dynamin domain, central interactive domain, GTPase effector domain, isoelectric point and localization in the corresponding isoforms are listed at the bottom.

To compare in detail the sequence similarities, the alignment of the three grass carp Mx sequences revealed that they each contained the characteristic Mx domains, i.e., the tripartite GTP-binding domain, the signature of the dynamin family, GTPase effector domain, and the LZ motif in the carboxyl terminal region. There are two LZ motifs in CiMx1 and CiMx3, but just one in CiMx2. No putative signal peptide was detected in any of them. Nuclear localization signals (NLS) and nuclear export signals (NES) were detected in CiMx1 and CiMx3. One potential N-linked glycosylation site was predicted in CiMx1, none in CiMx2, and two in CiMx3. The alignment also showed that the amino-terminal region of the proteins are highly conserved, whereas in the carboxyl-terminal region was poor conserved (Fig. 2), as has been found in other studies [7], [24].

**Figure 3:**
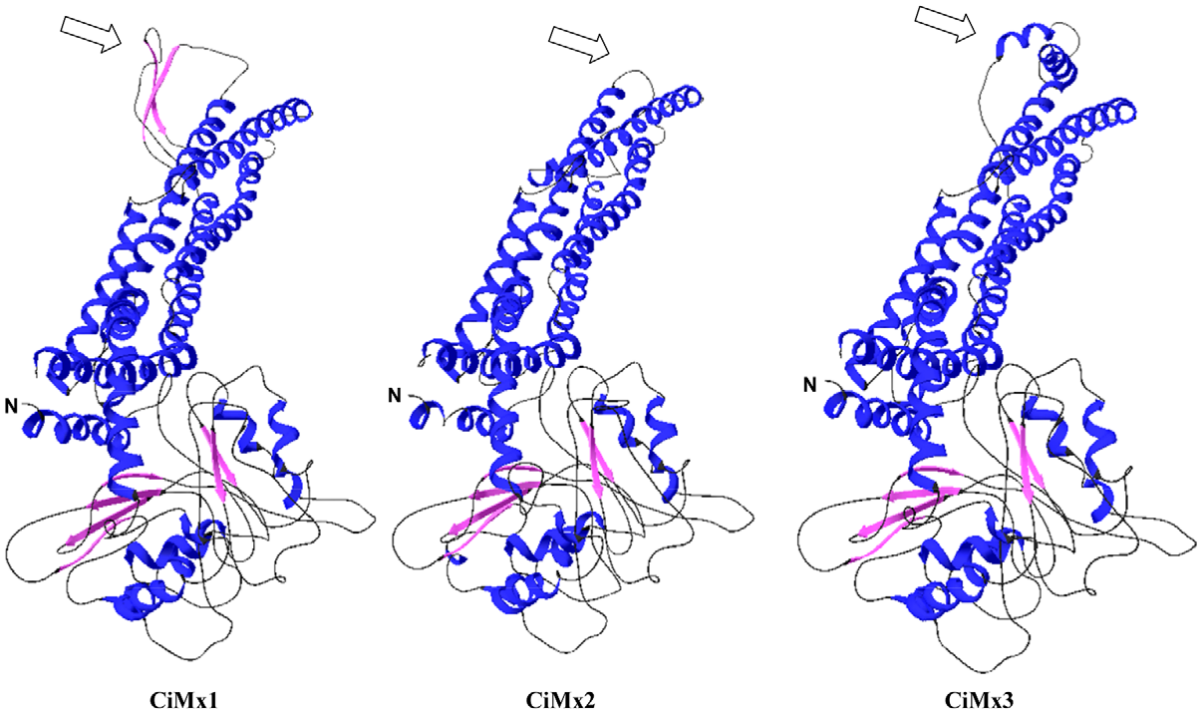
The spatial structures of three grass carp Mx isoforms predicted by SWISS-MODEL program. Blue, α-helices; pink, β-sheets; black, random coil. The hollow arrows mark the various structure among the three isoforms. In amino acid position 509–540, Glu517-Lys523 (overlapping with CID) and Leu534-Asp540 form two β-sheets in CiMx1; Ser509-Asn516 (overlapping with CID) forms α-helix in CiMx2; Ala512-Asp519 (overlapping with CID) and Thr525-Thr538 (overlapping with GED) form two α-helices in CiMx3.

To compare the structures of three grass carp Mx isoforms, tertiary structures were established using SwissModel Automatic Modelling Mode (Fig. 3). Surprisingly, the CiMx2 protein sequence has low homology with CiMx1 and CiMx3 (<50%, Table 1), and distributes into different groups (Fig. 1), but the structural difference occurs only in amino acids 509–540. In this region, there are two β-sheets in CiMx1, one α-helix in CiMx2, and two α-helices in CiMx3. All are located at the edge of the CID, which implies that although the basic functions remain conserved, the activities may vary due to differences in the structures.

### 3 Tissue distribution of grass carp Mx genes

**Figure 4:**
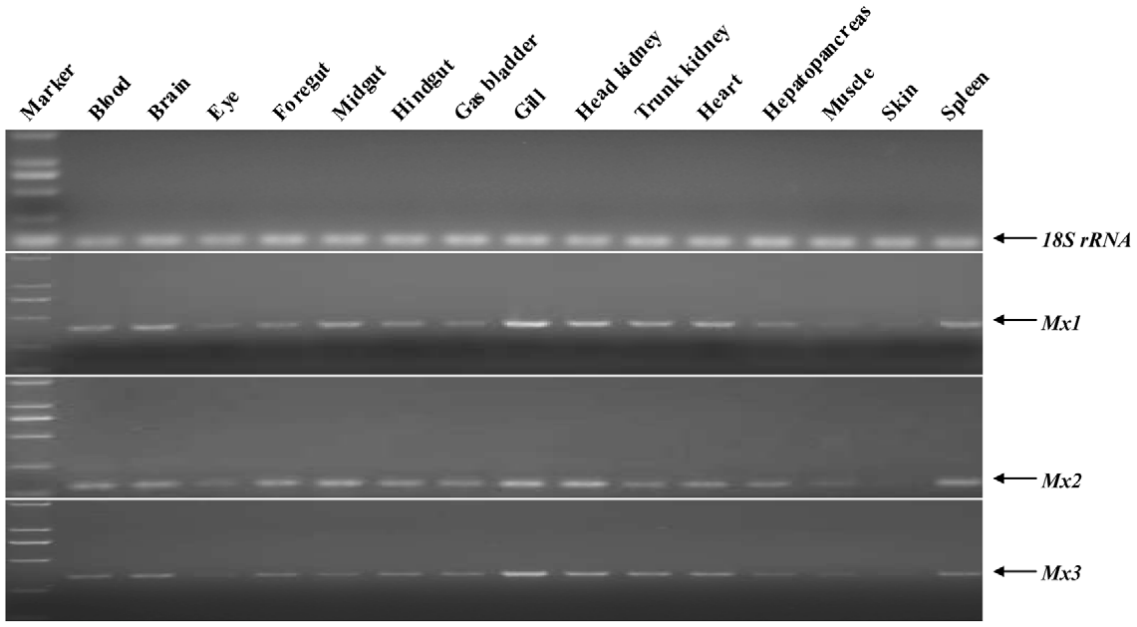
RT-PCR-based expression analysis of different Mx genes in various tissues of healthy grass carp. cDNAs from three animals for corresponding tissues were pooled for detection analysis. Reverse transcription and amplification by PCR with the specific primers were carried out for analyzing CiMx1, CiMx2, CiMx3 expression, and 18S rRNA was used as an internal reference. The 15 tested tissues are indicated above each lane. The top panel demonstrates 18S rRNA expression, and bottom three panels show the expression of different Mx genes. Gene names are indicated to the right of the panel.

The semi-quantitative RT-PCR (sqRT-PCR) technique was employed to determine Mx expression in blood, brain, eye, foregut, midgut, hindgut, gas bladder, gill, head kidney, trunk kidney, heart, hepatopancreas, muscle, skin and spleen. Mx mRNA expression was detected in all the examined tissues and was higher in gill and head kidney tissues, and lower in eye, muscle and skin tissues (Fig. 4). The tissue distribution of expression of the three genes was similar.

### 4 Response of grass carp Mx and IFN-I genes to GCRV infection in vivo

**Figure 5:**
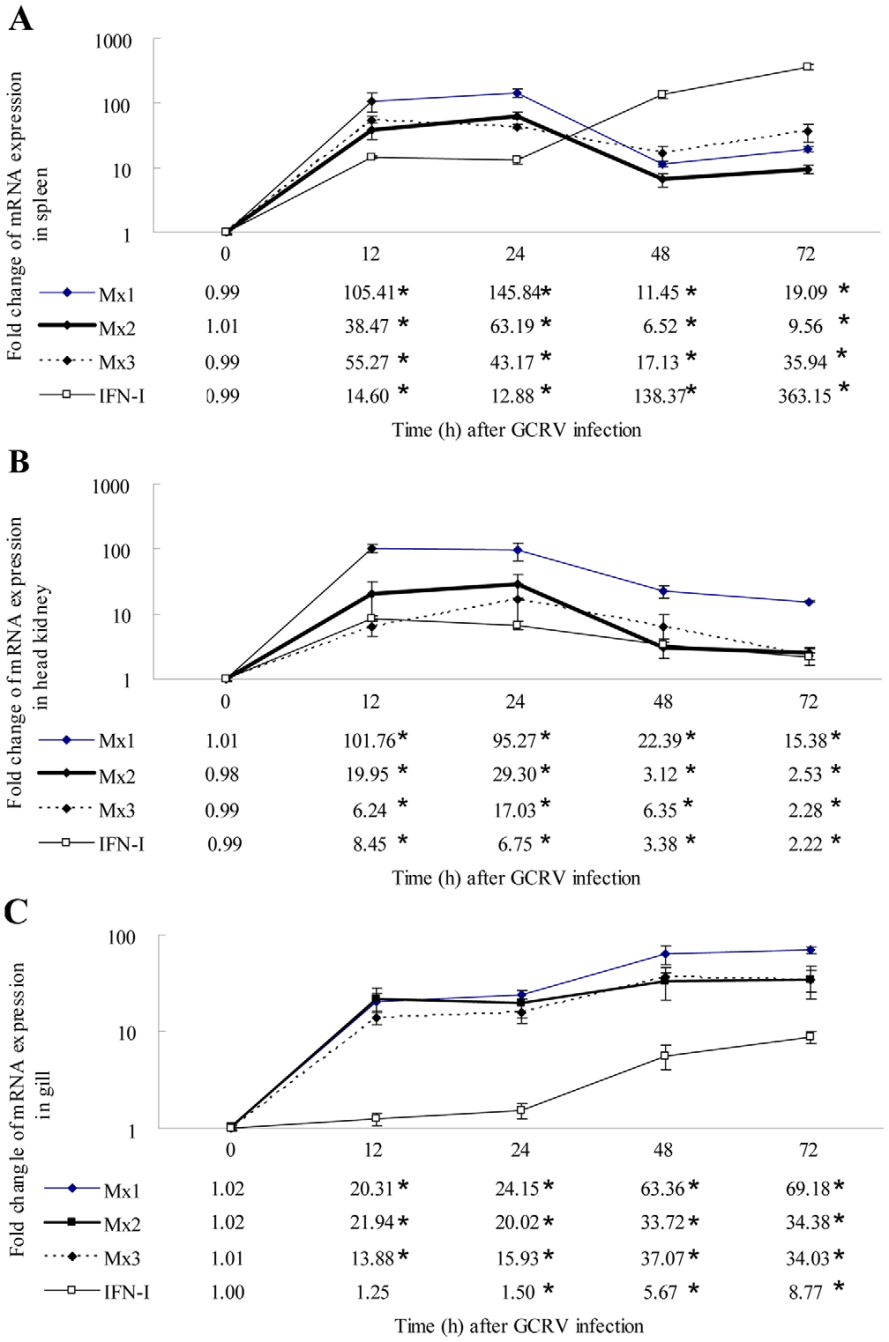
The mRNA expression profiles of three grass carp Mx genes and IFN-I gene post-GCRV injection in spleen, head kidney and gill tissues. 18S rRNA was employed as an internal control. A: spleen; B: head kidney; C: gill. Asterisks (*) mark the significant difference between experimental and control groups (P<0.05). Error bars indicate standard error.

The mRNA expression pattern of the grass carp Mx and IFN-I genes following GCRV infection was different in the three tissues (Fig. 5). In spleen, all three Mx mRNA was rapidly and significantly up-regulated at 12 h post-GCRV injection, quickly declined at 48 h, increased little at 72 h; however, IFN-I mRNA was continuously up-regulated (Fig. 5A). In head kidney, the transcripts of all the Mx and IFN-I genes were significantly up-regulated at 12 h, quickly declined at 48 h and 72 h; however, the extent of induction was lower than in spleen (Fig. 5B). In gills, the transcription of all the genes examined had a uniform trend; they were significantly up-regulated at 12 h or 24 h, and this trend lasted until the end of the experiment (Fig. 5C).

### 5 Regulation of grass carp Mx and IFN-I expressions to GCRV infection in vitro

**Figure 6:**
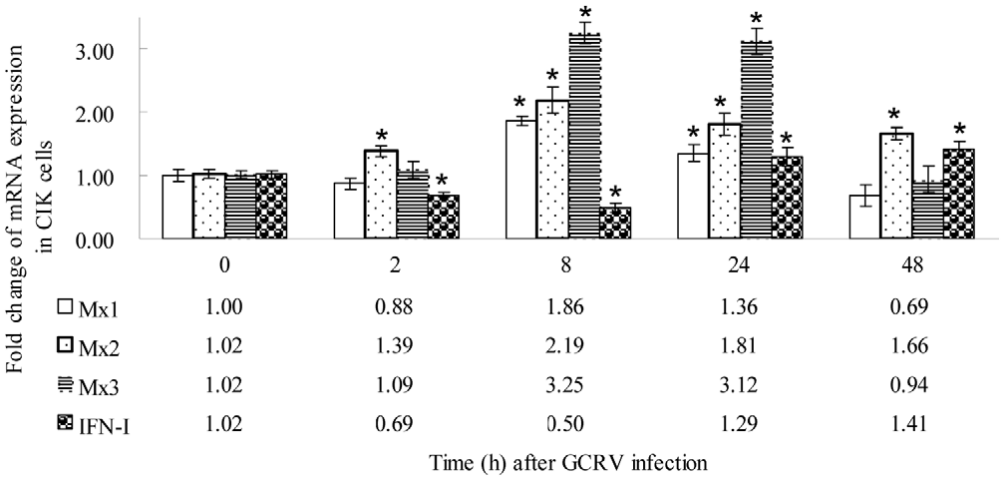
qRT-PCR-based expression analyses of different Mx genes and IFN-I gene in CIK cell culture. The cells were infected with GCRV and collected at different time-points (0, 2, 8, 24 and 48 h) after infection, then used in RNA extraction and qRT-PCR. EF1α was employed as an internal reference. Asterisks (*) mark the significant difference between experimental and control groups (p<0.05). Error bars indicate standard deviation.

In CIK cell culture, the mRNA expression profiles of the grass carp Mx and IFN-I genes were diverse after GCRV challenge (Fig. 6). CiMx1 and CiMx3 mRNA expression was up-regulated at 8 h post-challenge, and recovered to the normal level at 48 h. The CiMx2 transcript was elevated at 2 h, and remained at a higher level until the end of the experiment. CiIFN-I transcription was inhibited at 2 h, reached a trough at 8 h, and increased gradually.

### 6 Inducible expression of grass carp Mx and IFN-I genes by poly(I:C) stimulations

**Figure 7:**
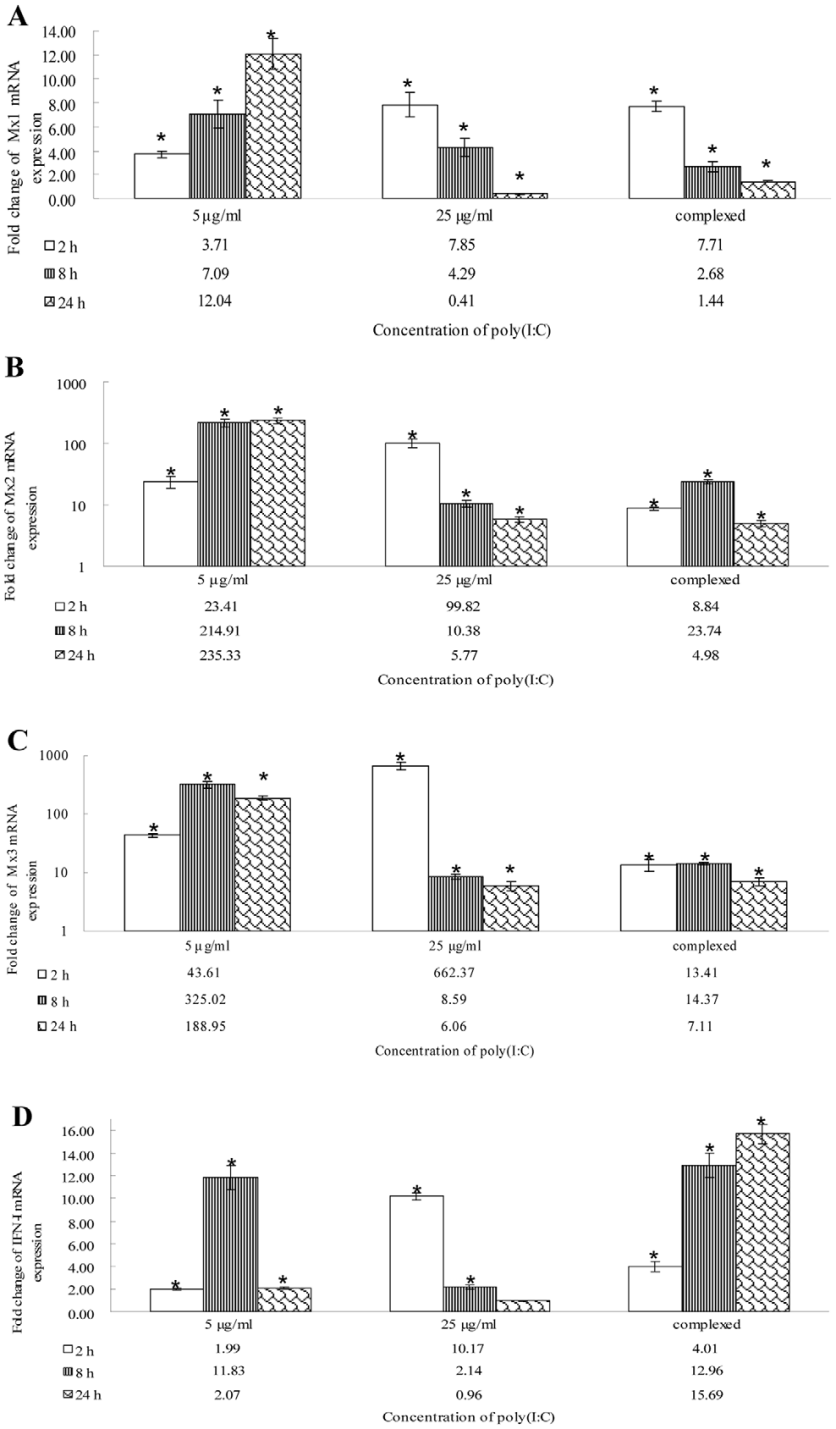
mRNA expression patterns of three grass carp Mx isoforms and IFN-I gene post poly(I:C) stimulation in CIK cells. EF1α was utilized as an internal control gene. The gene expression levels were measured at 2, 8 and 24 h post-stimulation. A: Mx1 transcription; B: Mx2 transcription; C: Mx3 transcription; D: IFN-I transcription. Asterisks (*) mark the significant difference between experimental and control groups (P<0.05). Error bars indicate standard error.

After stimulation with different concentrations of poly(I:C), Mx and IFN-I mRNA expression levels varied in CIK cells; CiMx1 and CiIFN-I responded weakly; and CiMx2 and CiMx3 responded strongly(Fig. 7). After stimulation with 5 µg/ml poly(I:C), CiMx1 and CiMx2 mRNA expression were up-regulated persistently; CiMx3 and CiIFN-I mRNA expression were up-regulated at 2 h post-stimulation, and regressed at 24 h. After stimulation with 25 µg/ml poly(I:C), all Mx and IFN-I transcripts were rapidely elevated at 2 h, and recovered gradually. After the complex stimulation, mRNA expression trends were different from those of naked 5 µg/ml of poly(I:C) stimulation.

### 7 Time course of expression of Mx and IFN-I genes in transformed cells

**Figure 8:**
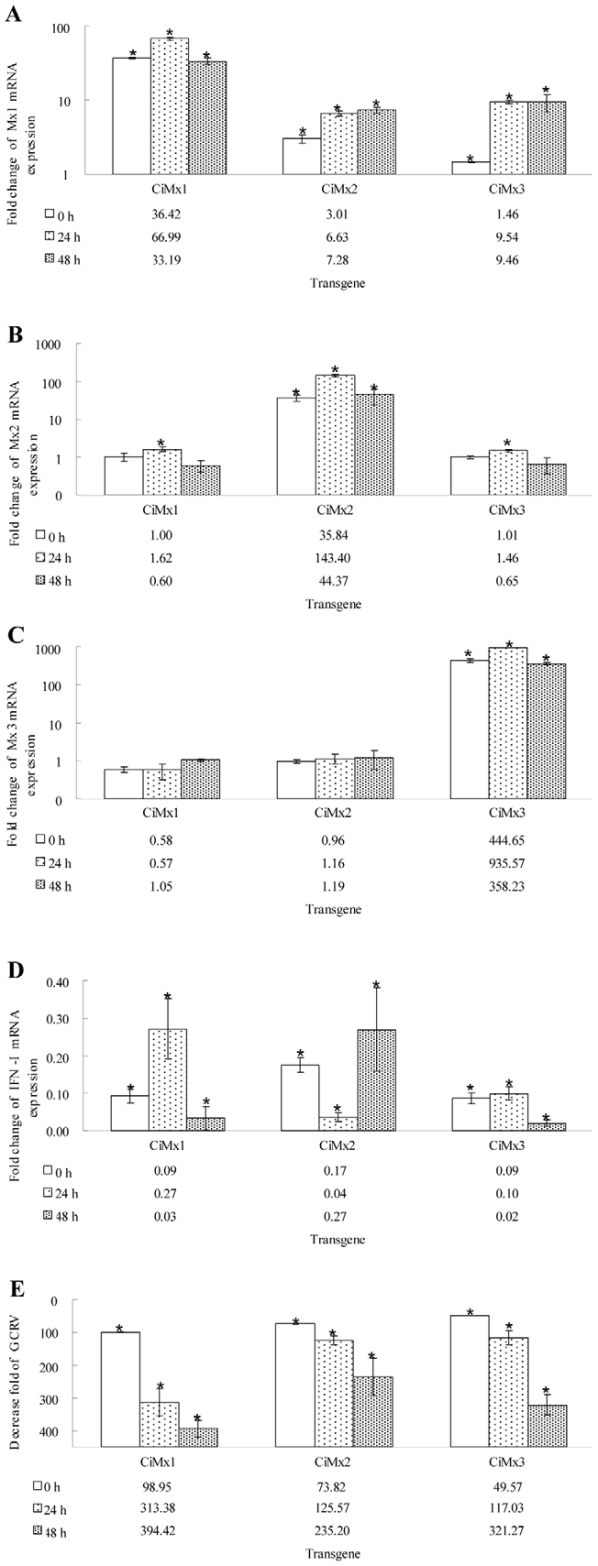
Analyses of the levels of three grass carp Mx, IFN-I and viral VP4 expression post-GCRV infection in transgenic cell lines. EF1α was used as an internal control gene. The gene expression levels were examined at 0, 24 and 48 h post-challenge. A: Mx1 expression levels; B: Mx2 expression levels; C: Mx3 expression levels; D: IFN-I expression levels; E: VP4 expression levels. Asterisks (*) mark significant differences between experimental and control groups (P<0.05). Error bars indicate standard error.

Stably transfected Mx-transgenic cell lines were infected with GCRV and then Mx and IFN-I mRNA expression levels were determined relative to control pCMV-eGFP transgenic cells. The transcription levels of endogenous Mx and the corresponding recombinant Mx were investigated. Mx1, Mx2 and Mx3 transcripts were substantially increased in the corresponding transgenic cell lines (Fig. 8A–C), and all displayed as similar pattern, first rising and then declining. The transcription of IFN-I was strongly feedback-inhibited in all the Mx-transgenic cells (Fig. 8D).

### 8 Effect of CiMx proteins on GCRV replication

**Figure 9:**
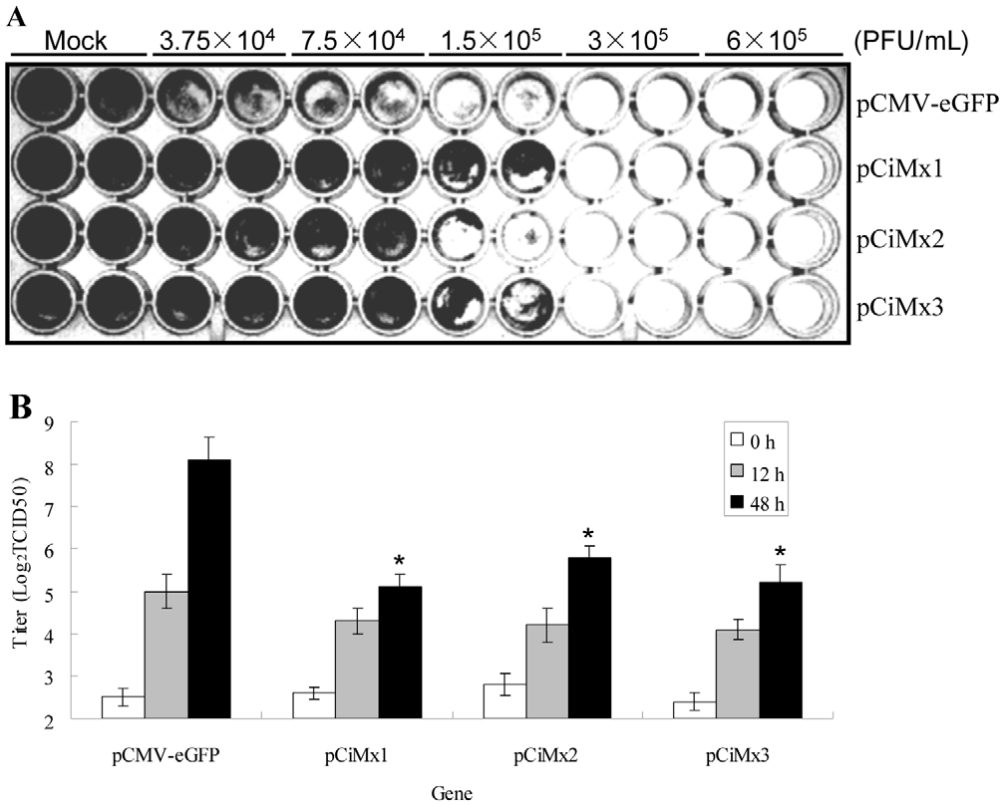
Antiviral activities of three grass carp Mx genes against GCRV in transgenic cells. Stable transgenic cells were cultured in 96-well plate for 24 h at 28°C. Monolayer cells were infected with GCRV in duplicate at the indicated densities. Cells were fixed with 4% paraformaldehyde and stained with 3% crystal violet at 60 h post-GCRV challenge (A). The culture supernatants from transgenic cells infected with GCRV were collected at 0 h, 12 h and 48 h post-infection, and the viral titers were determined for each culture by plaque assay in triplicate (B). Asterisks (*) mark significant differences between experimental and control groups (P<0.05). Error bars indicate standard error.

GCRV infection of CIK cells transfected with empty vector (pCMV-eGFP) led to complete cytopathic effect (CPE) at 60 h (Fig. 9A). In contrast, transfection of these cells with an expression vector encoding grass carp Mx (pCiMx1, pCiMx2, pCiMx3) protected cells against GCRV infection at 1.5×10^5^ PFU/ml (Fig. 9A). The ability to protect against GCRV was Mx1>Mx3>Mx2 (Fig. 9A). Over-expression of grass carp Mx decreased the GCRV titer in the cell culture at 24 h and 48 h post-GCRV infection, compared to the mock infected cells (Fig. 9B). Estimation of virus yields inside the cells by qRT-PCR indicated several-hundred-fold reduction compared to the control (pCMV-eGFP) (Fig. 8E).

## Discussion

Mx proteins are key components of the antiviral state induced by INFs in many species. They belong to the class of dynamin-like large GTPases known to be involved in intracellular vesicle trafficking and organelle homeostasis [11]. Generally, the N-terminal region is highly conserved and thought to have a regulatory function, whereas the C-terminal region is divergent and acts as an effector domain [25], which facilitates defense against a diverse range of viruses. The N-terminal GTPase domain binds and hydrolyses GTP [14], and the C-terminal LZ plays a role in the aggregation of dimers and trimers [26]. The tripartite GTP-binding domain (GXXXSGKS/T, DXXG and T/NKXD) and the dynamine family signature (LPRG(S/K)GIVTR) in the N-terminal region, as well as the putative LZ in C-terminal region, which are characteristic motifs found in all Mx proteins, also observed in grass carp Mx isoforms reported in this study (Fig. 2).

Piscine Mx sequences formed three branches (Fig. 1). The first branch contained a large number of piscine Mx sequences. The second branch was complicated, including Mx sequences from some of the family Cyprinidae, bird and mammals. The third branch only consisted of *D. rerio* MxG protein.

The three grass carp Mx isoforms were compared with each other:

1. The sizes of the three Mx protein sequences are similar (Fig. 2). The sequence identity between CiMx1 and CiMx3 is high (92.1%), and the identity is low with CiMx2 (49.2% for CiMx1, 49.5% for CiMx3 respectively) (Table 1).
2. CiMx1 and CiMx3 localize in the nucleus and cytoplasm, and CiMx2 exists in cytoplasm. No putative signal peptide was detected in any of them. NLS and NES were found in CiMx1 and CiMx3 (Fig. 2). The NLS is an amino acid sequence which ‘tags’ a protein for import into the cell nucleus by nuclear transport. There are two classical NLSs: monopartite NLSs having a single cluster of basic amino acid residues and bipartite NLSs having two clusters of basic amino acids separated by a 10–12 amino acid linker [27]. A putative consensus sequence of the bipartite NLS has been defined as (K/R)(K/R)X_10–12_(K/R)_3/5_ [28]. An NLS has the opposite function of a NES, which targets proteins for transportation out of the nucleus. The most common spacing of the hydrophobic residues in NESs has been found to be LxxxLxxLxL, where “L” is a hydrophobic residue (L, I, V, F, M) and “x” is any other amino acid [29]. Cotton rat Mx1 possesses NLS, but not in Mx2 [30]. Senegalese sole Mx lacks NLS [31], and Atlantic halibut Mx contains a putative bipartite NLS [32].
3. CiMx1 and CiMx3 are acidic proteins, and CiMx2 is a neutral protein. The isoelectric points are 5.37, 5.29, and 7.05 for CiMx1, CiMx3, and CiMx2, respectively (Fig. 2). Acidic proteins tend to be degraded faster than neutral or basic ones [33].
4. CiMx1 and CiMx3 contain two LZ motifs, and there is only one in CiMx2 (Fig. 2). LZ represents a characteristic property of DNA binding proteins, facilitating dimerization [34] and is also essential for antiviral activity [35].
5. One potential N-linked glycosylation site (NXT/S) was found in CiMx1, zero in CiMx2, and two in CiMx3 (Fig. 2). No N-linked glycosylation site is observed in the sea bream Mx sequence [36], one in the rainbow trout Mx2 sequence, and two in the rainbow trout Mx1 and Mx3 sequences [37]. N-linked glycosylation is important for the folding of some eukaryotic proteins [38].
6. The 3D structures are different at the edge of CID and GED (Fig. 3). The structural difference in Mx proteins has not attracted attention from other scientists, which implies a variety of functions.
7. The sizes and putative features of full-length cDNAs of grass carp Mx genes vary greatly among each other. The CiMx1, CiMx2 and CiMx3 genes consist of 2881, 2367 and 3002 nucleotides respectively, although the ORFs are similar in size. The eukaryotic polyadenylation consensus motif (AATAAA) is present in all the three grass carp Mx genes. It also occurs in Japanese flounder Mx [39] but not in sea bream Mx [36]. In metazoans, cleavage and polyadenylation occurs 10–30 nucleotides 3′ of the hexamer motif (AATAAA/ATTAAA) [40]. Two mRNA instability motifs (ATTTA) were observed in CiMx1, three in CiMx2, and seven in CiMx3. Unstable mRNAs often contain an instability motif in the 3′ UTR, and this is especially true for inflammatory-related mRNAs [41]. The results indicated the mRNA stability is significantly different in the three grass carp Mx genes.

Taken together, CiMx2 exerts functions in cytoplasm, whilst CiMx1 and CiMx3 play roles in cytoplasm and nucleus. CiMx1 and CiMx3 share high amino acid sequence identity (92.1%); however, unstable CiMx3 mRNA, various 3D structures and the N-linked glycosylation site might infer that CiMx3 has evolved supplementary functions to CiMx1 under selective pressure.

Mx genes have widespread tissue distribution (Fig. 4), which indicates they play roles in multiple tissues. In other fishes, Mx genes are also ubiquitous in different tissues, such as sea bream [36] and Japanese flounder [39].

After GCRV injection, mRNA expression profiles of IFN-I and its effector Mx genes are different from each other in different tissues (Fig. 5). IFN-I transcripts rose continuously and largely in spleen, continuously and diminutively in gill, and rose a little and declined in head kidney. The transcription of Mx isoforms rose greatly and declined in spleen and head kidney, rose continuously in gill, but the extent varied for the three Mx genes. The results suggested Mx mRNA expression may be independent of IFN-I. In two rainbow trout cell lines: monocyte/macrophage RTS1 and fibroblast-like RTG-2, poly(I:C) and chum salmon reovirus (CSV) induce Mx transcripts in the presence of cycloheximide, suggesting a direct induction mechanism (independent of IFN) is also possible [42]. The IFN transcripts are induced later than Mx transcription in the infectious salmon anemia virus (ISAV) infected cells indicated that Mx is induced through IFN-independent pathways in the early stages of ISAV infection [43].

Following GCRV infection *in vitro*, gene expression was subtly examined at early stages. IFN-I and Mx mRNA expression trends were consistent with those in spleen tissue according to the data at same time points (Fig. 5A and Fig. 6). The results showed CIK cells are suitable for antiviral studies *in vitro*, although the specific values are not constant. The expression kinetics of the three rainbow trout Mx genes are differently regulated *in vitro* and *in vivo* [44]. Chicken Mx1 responses *in vivo* may differ markedly from those observed *in vitro* [45].

After stimulation with different concentrations of synthetic dsRNA poly(I:C), distinct kinetics of IFN-I and Mx gene transcription appear in a dose-dependent and time-dependent manners (Fig. 7). Compared with the mRNA expression patterns post-GCRV challenge (Fig. 6), the induced responses are faster and more intense after poly(I:C) stimulation, indicating that GCRV strongly blocks expression of these genes. By qRT-PCR, the extent and timing of brown trout Mx expression was shown to differ following treatment with poly(I:C) and single or dual viral infections [46].

In stable transgenic cells, all the corresponding Mx genes were over-expressed, and transcription was induced by GCRV challenge (Fig. 8A–C). In general, the expression of Mx genes is tightly regulated by the presence of INF-I [30]. Interestingly, the expression of IFN-I was strongly feedback-inhibited in the transgenic cell culture (Fig. 8D), suggesting that antiviral responses must be tightly regulated in order to prevent uncontrolled production of type I IFN that might have deleterious effects on the host [47].

Mx proteins are IFN-induced proteins that are widespread in eukaryotes, however, their antiviral activity is unclear and the results variable among species [48]. Therefore, assessment of the putative Mx antiviral activity in each species is of interest. The Mx-over-expressing cells delayed the appearance of CPE after infection by GCRV (Fig. 9A). Compared with controls, the yield of GCRV was reduced several-hundred-fold in the Mx-transgenic cells (Fig. 8E). All titers increased at 48 h more than those at 12 h post-infection, but they were reduced greatly compared to control cells at the corresponding time points (Fig. 9B). These results demonstrate all the three Mx isoforms possess substantial antiviral activity. Over-expression of rare minnow Mx can improve survival after GCRV infection [49]. The expression of barramundi Mx gene is also able to inhibit the proliferation of fish nodavirus and birnavirus [50]. Barramundi Mx suppresses viral RNA synthesis by interaction with viral RNA-dependent RNA polymerase (RdRp), and redistributes RdRp to the perinuclear area for degradation [51]. Grouper Mx over-expression has an inhibitory effect on nodavirus coat protein and RdRp, which results in reduced viral yields [52]. Human Mx1 may form oligomeric rings around tubular nucleocapsid structures, thereby inhibiting their transcriptional and replicative function [14].

However, some Mx proteins lack antiviral activity. Expression of Mx proteins in a chinook salmon cell line did not interfere with accumulation of infectious haematopoietic necrosis virus (IHNV) nucleoprotein [37]. Mx is dispensable for interferon-mediated resistance of chicken cells against influenza A virus [53]. The viral nucleoprotein determines Mx sensitivity of influenza A virus [54]. There is even an inverse relationship between the levels of Mx and infectious pancreatic necrosis virus (IPNV) in Atlantic salmon parr [55]. In summary, antiviral activity of Mx proteins is diverse and complicated.

## Materials and Methods

### Ethics statement

No specific permit is required for experimental use of grass carp or GCRV in Shaanxi Province, China. Neither is privately-owned nor protected in any way. The studies did not involve endangered or protected species. This study has been reviewed and approved by the ethics committee of Northwest A&F University.

### Animal, viral infection and sample collection

Grass carp (average weight 15–20 g) were collected from Ankang Aquatic Animal Experiment and Demonstration Station of Northwest A&F University (Ankang, China) and were acclimatized to laboratory conditions for one week in a quarantine area by maintenance in 300 L aerated aquaria at 28°C, fed twice daily.

For tissue distribution analysis of mRNA expression, tissues including blood, brain, eye, foregut, midgut, hindgut, gas bladder, gill, head kidney, trunk kidney, heart, hepatopancreas, muscle, skin and spleen were sampled from three healthy grass carp.

For the viral challenge experiment, 100 μL of GCRV (097 strain, 3.63×10^7^ TCID_50_/ml in PBS) per gram body weight was injected intraperitoneally. Control animals were injected with PBS. Five individuals were sacrificed and tissues including spleen, head kidney and gill were harvested at 0, 12, 24, 48, 72 h post-injection.

The samples were homogenized in TRIZOL reagent (Invitrogen) and total RNAs were isolated according to the manufacturer's instruction. Total RNAs were incubated with RNase-free DNase I (Roche) to eliminate contaminating genomic DNA before being reverse-transcribed into cDNA using random hexamer primers and M-MLV reverse transcriptase (Promega).

### Cell, immune challenge and sample preparation

*C. idella* kidney (CIK) cells, provided by China Center for Type Culture Collection, were grown in medium DMEM-F12 supplemented with 10% inactivated fetal calf serum (Gibco BRL), 100 U/ml of penicillin and 100 U/ml of streptomycin sulfate. Cells were maintained at 28°C in 6-well culture plates. All the following stimulations or infections were performed in four parallel wells.

For virus infection, CIK cells were infected with GCRV at a multiplicity of infection (MOI) of 1. The control was treated with PBS. For time-dependent expression profiles, the cells were collected at 0, 2, 8, 24, 48 h post-infection. RNA was extracted and reverse transcribed.

For synthetic dsRNA stimulation, polyinosinic-polycytidylic potassium salt (poly(I:C)) (Sigma-Aldrich, St. Louis, MO, USA) dissolved in PBS was heated to 55°C for 5 min and allowed to cool to room temperature. For naked poly(I:C) treatment, 1×10^6^ cells were treated with 5 µg/ml and 25 µg/ml (final concentration) of poly(I:C). Complexed poly(I:C) treatment was performed by transfecting 5 µg/ml of poly(I:C) (final concentration) coupled to Lipofectamine 2000 (Invitrogen). The control was treated with PBS. For kinetic studies, cells were harvested at 2, 8, 24 h post-stimulation, centrifuged at 1000 rpm for 6 min. RNA was isolated and reverse transcribed.

### Construction of cDNA library and EST analysis

A cDNA library was constructed with CIK cells post-GCRV infection, using the Creator SMART cDNA Library Construction Kit (Clontech). Random sequencing of the library using T7 primer obtained 10228 successful sequencing reactions. BLAST analysis of all the 10228 EST sequences revealed that two different ESTs (CIK10130, 649 bp; and CIK07320, 567bp) were homologous to *C. auratus* Mx1 (GenBank accession No. **AY303813**). Based on the homology, they were named CiMx1 and CiMx3. The EST sequences were then selected for further cloning of the full-length CiMx1 and CiMx3 cDNA.

### Cloning of the full-length CiMx1 and CiMx3 cDNA

According to the EST sequences of clone CIK10130 and clone CIK07320, the 3′ and 5′ ends of CiMx1 and CiMx3 cDNAs were obtained by RACE (rapid amplification of cDNA ends) technique. RACE was carried out using the BD SMART^TM^ RACE cDNA amplification kit (BD Biosciences Clontech) and 5′ RACE system (Invitrogen). The first-strand cDNA synthesis and RACE were performed on spleen-derived RNA. To obtain the 3′ region of CiMx1, primer pairs MF328a/adaptor primer UPM and MF329a/adaptor primer NUP (Table 2) were used for primary PCR and nested PCR, respectively. To obtain the 3′ end of CiMx3, specific primers MF328 and MF329 (Table 2) were used for the primary and nested PCRs respectively. Similarly, the 5′ end of CiMx1 was obtained by nested PCR using primer pairs MR347a/AAP and MR348a/AUAP (Table 2), and MR347b/AAP and MR348b/AUAP (Table 2) for CiMx3. The full-length cDNA sequences were confirmed by sequencing the PCR products amplified by primers MF371a and MR372a for CiMx1 and primers MF371a and MR372b (Table 2) for CiMx3, within the predicted 5′ and 3′ untranslated regions (UTRs) respectively. All the PCR products were gel-purified, cloned into pMD18-T Easy vector, transformed into *Escherichia coli* TOP10 competent cells, and plated on an LB-agar petri-dish with ampicillin. Positive colonies containing inserts of the expected size were screened by colony PCR and three were picked and sent to a commercial company (Genscript Biotechnology Co., Ltd, China) for sequencing.

**Table 2:** Primer sequences and their designated applications in this study.

Primer name	Sequence (5′→3′)	Primer usage and amplicon length (nt)
CiMx1		
MF328a	GCAAGCGTCTAGCTGACCAG	3′ RACE
MF329a	GCAAGATAAAGACGGTGTAGACAT	
MR347a	ATTTTCAGAAGATTAGGGAATCCAATA	5′ RACE
MR348a	CTTCTTCCTAACCACATCAGAGACA	
MF371a	TGAGGAAAAAATCCGCCC	Sequence confirmation 2623
MR372a	CATTGCTTGAGAAAAAA	
MF432	AACTGgctagcTTTCCGACGTTGTTTAAGG	Expression vector 2003
MR433	AACTGgggcccGCTCATGTTTTTCATTGCC	
MF426	CTGGGGAGGAAGTAAAGTGTTCT	qRT-PCR 392
MR427	CAGCATGGATTCTGCCTGG	
CiMx2		
MF434	AACTGgaattcCACATAGGCGTCGCTGGTG	Expression vector 2020
MR435	AACTGgggcccCAAGACAGGTCCTAAATGACAAACT	
MF428	ACATTGACATCGCCACCACT	qRT-PCR 129
MR429	TTCTGACCACCGTCTCCTCC	
CiMx3		
MF328	GCAAGCGTCTATCTGACCAG	3′ RACE
MF329	TCCCAATGGTGATCCGCTATC	
MR347b	ATTTTCAGAAGATTAGGGAATCCAGCG	5′ RACE
MR348b	CTTCCTTCTAACCACATCGGCGATG	
MF371a	TGAGGAAAAAATCCGCCC	Sequence confirmation 2738
MR372b	AATCACAATCAATTAAAAG	
MF432	AACTGgctagcTTTCCGACGTTGTTTAAGG	Expression vector 2037
MR436	AACTGgggcccTATGAGGCGAGAGTGCATG	
MF430	CCTTAAAGACGCTGAAGACCA	qRT-PCR 342
MR431	GCAACCTCATCTCACGCAA	
CiIFN-I		
IF590	AAGCAACGAGTCTTTGAGCCT	qRT-PCR 79
IR591	GCGTCCTGGAAATGACACCT	
*VP4*		
VF146	CGAAAACCTACCAGTGGATAATG	Virus detection and qRT-PCR 135
VR147	CCAGCTAATACGCCAACGAC	
18S rRNA		
18F99	ATTTCCGACACGGAGAGG	qRT-PCR 90
18R100	CATGGGTTTAGGATACGCTC	
EF-1a		
EF125	CGCCAGTGTTGCCTTCGT	qRT-PCR 99
ER126	CGCTCAATCTTCCATCCCTT	
CMV promoter		
MF552	ATctcgagTAGTTATTAATAGTAATCAATTACG	Introducing CMV promoter 620
MR553a	ACTGaagcttgggcccgctagcCGATCTGACGGTTCACTA	
MR554a	ACTGaagcttgggcccgaattcCGATCTGACGGTTCACTA	
SV40 transcriptional termination site		
SVF81a	ACTGgggcccAGCGGCCGCGACTCTAGATCAT	Transcriptional termination 224
SVR95a	ACTGaagcttGCAGTGAAAAAAATGCTTTATTTGTG	
pCMV-eGFP		
S579	CCCTGATTCTGTGGATAACCG	Sequencing CMV promoter
3′-RACE universal adaptor primer		
UPM	Long: CTAATACGACTCACTATAGGGCAAGCAGTGGTATC AACGCAGAGT Short: CTAATACGACTCACTATAGGGC	3′ RACE
NUP	AAGCAGTGGTATCAACGCAGAGT	
5′-RACE adaptor primer		
AAP	GGCCACGCGTCGACTAGTACGGGIIGGGIIGGGIIG	5′ RACE
AUAP	GGCCACGCGTCGACTAGTAC	

### Sequence analysis

The searches for nucleotide and protein sequence similarities were conducted using the BLAST algorithm at the National Center for Biotechnology Information (http://www.ncbi.nlm.nih.gov/blast) and Matrix Global Alignment Tool (MatGAT) (http://bitincka.com/ledion/matgat/). The deduced amino acid sequence was analyzed with the Expert Protein Analysis System (http://www.expasy.org/). The protein domains were determined by Simple Modular Architecture Research Tool (SMART) (http://smart.embl-heidelberg.de/) and Pfam database search (http://pfam.sanger.ac.uk/search). Potential glycosylation sites were predicted by a scan of the sequence against the PROSITE database (http://us.expasy.org/tools/scanprosite). NLS and NES were predicted by putative patterns and NetNES 1.1 Server (http://www.cbs.dtu.dk/services/NetNES/) [28], [29]. Phylogenetic and molecular evolutionary analysis was conducted using MEGA (Molecular Evolutionary Genetics Analysis) version 5.1 and optimized manually. Multiple sequence alignments were performed using the ClustalW Multiple Alignment program (http://www.ebi.ac.uk/clustalw/) and Multiple Alignment display program (http://www.bioinformatics.org/sms/). The three-dimensional (3D) structure was determined using the SWISS MODEL prediction algorithm (http://swissmodel.expasy.org/) and displayed by using the DeepView program.

### Quantification of gene expression

Real-time fluorescence quantitative reverse transcription polymerase chain reaction (qRT-PCR) was established in a CFX96™ Real-Time PCR Detection System (Bio-Rad), to quantify CiIFN-I, CiMx1, CiMx2, CiMx3 and GCRV gene expression *in vivo* and *in vitro*. 18S rRNA and EF1α genes were employed as internal control genes for cDNA normalization *in vivo* and *in vitro* assays [56], respectively. The primers used in the qRT-PCR are listed in Table 2. The qRT-PCR mixture consisted of 2 µl (400 ng) of cDNA sample, 7.6 µl nuclease-free water, 10 µl of 2× SYBR Green PCR master mix (TaKaRa), and 0.2 µl of each gene specific primer (10 µM). The PCR cycling conditions were: 1 cycle of 95°C/30 s, 40 cycles of 95°C/5 s, 60°C/30 s, 1 cycle of 95°C/15 s, 60°C/30 s, 95°C/15 s, followed by dissociation curve analysis (65°C to 95°C: increment 0.5°C for 5 s) to verify the amplification of a single product. The threshold cycle (CT) value was determined using the manual setting on the CFX Sequence Detection System and exported into a Microsoft Excel Sheet for subsequent data analyses where the relative expression ratios of target gene in treated group versus those in control group were calculated by 2^−ΔΔCT^ method. The expression data obtained from the independent biological replicates were subjected to one-way analysis of variance (one-way ANOVA), followed by an unpaired, two-tailed t-test. Differences were considered statistically significant when P<0.05.

### Tissue distribution of CiMx1, CiMx2 and CiMx3 mRNA

To intuitionistically show CiMx1, CiMx2 and CiMx3 mRNA expressions in different tissues, sqRT-PCR was employed. Three cDNAs from three animals for each tissue were equally pooled for tissue distribution analysis to minimize individual variability. 18S rRNA served as an internal reference gene to normalize mRNA expression in different tissues [56]. All primers were the same as those in the qRT-PCR trials (Table 2). sqRT-PCR was carried out in a volume of 25 µl containing 1 µl (100 ng) of cDNA as template, 2.5 µl of 10× PCR buffer, 2.5 µl of MgCl_2_ (2.5 mM), 0.5 µl of each primer (10 µM), 1 µl of dNTP (10 mM), 16.8 µl of PCR-grade water, and 0.2 µl (1U) of Taq polymerase (MBI, Fermentas). The PCR run 20 cycles for 18S rRNA and 32 cycles for three Mx genes. All the PCR products were separated by electrophoresis on 1% horizontal agarose gel.

### mRNA expression patterns of CiMx1, CiMx2, CiMx3 and CiIFN-I genes in spleen, head kidney and gill tissues after GCRV challenge

To determine the effects of viral infection on CiMx1, CiMx2, CiMx3 and their inducer CiIFN-I in individual level, three representative tissues were employed. Spleen is the putative major innate immune tissue in fish and head kidney is another major piscine immune tissue. According to the above tissue distribution analysis, all the three Mx isoforms expressed highest in the gill tissue, and gill is directly exposed in the aquatic environment containing a huge microbial biomass. Five animals were sacrificed at 0, 12, 24, 48, 72 h after GCRV or PBS injection, and head kidney, spleen and gill tissues were collected. qRT-PCR was performed to determine the mRNA expression pattern.

### mRNA expression profiles of CiMx1, CiMx2, CiMx3 and CiIFN-I genes in CIK cell culture following GCRV infection

To examine the influence of GCRV on CiMx1, CiMx2, CiMx3 and CiIFN-I *in vitro*, the CIK cell line was employed. Samples were collected at 0, 2, 8, 24 48 h after GCRV infection and mRNA expression was determined by qRT-PCR.

### mRNA expression levels of CiMx1, CiMx2, CiMx3 and CiIFN-I genes in CIK cells post poly(I:C) stimulation

To compare CiMx1, CiMx2, CiMx3 and CiIFN-I mRNA expression post viral challenge and after synthetic analog of dsRNA stimulation, CIK cells were stimulated by different concentrations of poly(I:C), and samples were collected at 2, 8, 24 h post-treatment. The corresponding gene expression levels were determined by qRT-PCR.

### Construction of expression cassettes

pCMV-eGFP (Clontech, USA) was used as the original plasmid. A second CMV promoter was introduced at restriction sites by PCR, using the pCMV-eGFP as template. Sense primer was MF552a with an *Xho*I site, and antisense primer was MR553a with *Bmt*I, *Apa*I, and *Hin*dIII sites for CiMx1 and CiMx3 vectors, whereas the antisense primer was MR554a with *Eco*RI, *Apa* I, and *Hin*dIII sites for CiMx2 vector (Table 2). The PCR products were digested with *Xho*I and *Hin*dIII and plasmid pCMV-eGFP was also digested with the same enzymes. The target fragments were purified, ligated with T4 ligase and named pCMV-eGFP-CMVs, which were sequenced with primer S579 to verify inserts (Table 2).

The SV40 transcriptional termination site was introduced by PCR using plasmid pCMV-eGFP as template. The forward primer was SVF81a with an *Apa*I site, and the reverse primer was SVR95a with a *Hin*dIII site (Table 2). The amplicon was ligated into pMD18-T Easy vector and confirmed by sequencing. The plasmid with the correct insert was digested with *Apa*I and *Hin*dIII, and cloned into plasmids pCMV-eGFP-CMVs. They were designated with pCMV-eGFP-CMV-SV40s.

The CiMx open reading frame (ORF) was amplified by sense primer MF432 with a *Bmt*I site and antisense primer MR433 with an *Apa*I site for CiMx1, and MF432 and MR436 for CiMx3 (Table 2), using the reverse transcription product for 3′ RACE as a template, respectively. The amplicons were ligated into pMD18-T Easy vector and confirmed by sequencing. The plasmids with the target sequence were digested with *Bmt*I and *Apa*I, and inserted into plasmid pCMV-eGFP-CMV-SV40, respectively. The recombinant plasmids were designated pCMV-eGFP-CMV-Mx1-SV40 (abbreviation, pCiMx1) and pCMV-eGFP-CMV-Mx3-SV40 (abbreviation, pCiMx3), respectively.

For CiMx2, the primer set was sense primer MF434 with an *Eco*RI site and antisense primer MR435 with an *Apa*I site (Table 2). The amplicon was handled and verified as above. The plasmid with target sequence was digested with *Eco*RI and *Apa*I, and inserted into plasmid pCMV-eGFP-CMV-SV40. The recombinant plasmid was named pCMV-eGFP-CMV-Mx2-SV40 (abbreviation, pCiMx2).

### Transfection of expression constructs and establishment of stable cell lines

Expression constructs were introduced into CIK cells by FuGENE® HD Transfection Reagent (Roche) according to the manufacturer's instructions. Briefly, CIK cells were seeded in 12-well culture plates with 1×10^6^ cells per well, and incubated at 28°C for 24 h. Once cells reached approximately 80% confluence, they were washed with PBS, and then the culture supernatant was replaced with Opti-MEM (Invitrogen). Cells were transfected with 500 ng of each expression construct (pCiMx1, pCiMx2, pCiMx3 and pCMV-eGFP) and 6 μl of FuGENE® HD Transfection Reagent (Roche). At 24 h post-transfection, cells were examined under fluorescence microscope.

The G418 screening method was used for establishment of stable cell lines. In short, when GFP in transfected cells emerged, 400 μg/ml of G418 was supplied. The medium was changed every two days. Approximately two weeks later, when positive cells reached 50%, they were employed for the following experiments.

### GCRV challenge, gene expression analyses and virus quantification

After stable transgenic cell lines were obtained, they were inoculated into 24-well culture plates and infected with GCRV at an MOI of 1. Samples were collected at 0, 24, 48 h post-infection. Total RNA was isolated and cDNA was prepared as above. CiMx1, CiMx2 CiMx3 and CiIFN-I mRNA expression was examined by qRT-PCR. The GCRV VP4 gene transcript was detected by qRT-PCR to quantify the virus yield.

### Antiviral activity assays

To study whether over-expression of the three grass carp Mx isoforms could induce an antiviral state in cells, an antiviral assay against GCRV was performed using the above transgenic cells. Cells were transferred into a 96-well plate at a density of 4×10^5^ cells/well. Cell monolayers were infected with GCRV at the indicated densities (Fig. 9A) in duplicate. After 60 h of culture, cells were washed with PBS, fixed with 4% paraformaldehyde (PFA) for 15 min, stained with 3% crystal violet for 15 min, washed three times with distilled water and allowed to air dry. The image was taken under Bio Rad imaging system.

500 μl supernatants in 24-well plates containing transgenic cells were harvested at 12 and 48 h post-GCRV infection and subjected to viral titration assay. Virus titers were determined by the 50% tissue culture infective dose (TCID_50_) method. In brief, viral titration was performed in CIK cells in 96-well plates at a density of 4×10^5^ cells/well. Two-fold serial dilutions of the supernatants were then added to the monolayers. At three days post-infection, the cells were fixed with 4% PFA and stained with 3% crystal violet for calculating the virus titers. Three independent experiments were carried out for each treatment.
